# Mapping Aural Rehabilitation Needs in a Kannada-Speaking Population: A Focus Group Study with Adults with Hearing Loss, Their Significant Others and Audiologists

**DOI:** 10.3390/audiolres16030087

**Published:** 2026-06-04

**Authors:** Gudambe Nellithaya Spoorthi, Usha Shastri, Kaushlendra Kumar, Mohan Kumar Kalaiah

**Affiliations:** Department of Audiology and Speech Language Pathology, Kasturba Medical College Mangalore, Manipal Academy of Higher Education, Manipal 576104, India; spoorthi.mchpmlr2023@learner.manipal.edu (G.N.S.);

**Keywords:** aural rehabilitation, focus group discussion, informational counseling, adult with hearing loss, significant others

## Abstract

**Background/Objectives**: The present study aimed to verify the importance of different components of aural rehabilitation and evaluate their relevance and acceptability among the Kannada-speaking population in India. This evaluation is intended to inform the development of a tailored aural rehabilitation module for this population. **Methods**: A thorough literature review was conducted to gain insights into the components of aural rehabilitation in the Western context. The insights gathered from this review served as foundational information for a focus group discussion. The focus group discussion consisted of 15 participants, including audiologists, adults with hearing loss, and their significant others. The data were transcribed and analyzed using inductive thematic analysis. **Results**: The audiologists in our study confirmed that the components of aural rehabilitation presented in the literature are essential, including sensory management, informational counseling, perceptual training, and personal adjustment counseling. Perspectives of significant others are the highlight of this study as they are less explored in the context of aural rehabilitation in India. Other themes identified from the FGDs included the involvement of family, the impact of hearing loss, awareness of various management options, and challenges/barriers related to aural rehabilitation. Furthermore, most of the adults with hearing loss and their significant others primarily viewed hearing aids as the sole solution. **Conclusions**: This study highlights the importance of a comprehensive aural rehabilitation program for adults with hearing loss and their significant others in the Kannada speaking population. It emphasizes the importance of informational counseling, communication strategies, and psychosocial support. Involving significant others might foster understanding and support, aiding in the acceptance of hearing loss and its rehabilitation, ultimately improving everyday communication and addressing functional challenges.

## 1. Introduction

Hearing loss is a common issue among older adults, with approximately one in four individuals above 60 years being affected by it [1]. This greatly affects older adults’ daily activities [2], increases the chance of falls [3], reduces physical activity [4], leads to depression [5], reduces social interaction and affects cognitive function. It can also lead to cognitive decline and even dementia [6,7]. Adults with hearing loss (AHL) often have difficulty communicating, particularly under noisy or reverberant conditions [8]. Hearing loss can negatively impact their quality of life (QoL) [9,10]. Additionally, the impact of hearing loss extends beyond the individuals affected, as significant others (SOs) also feel as much anxiety and frustration as the AHLs themselves do [11,12]. Significant others face various activity limitations and participation restrictions because of their partner’s hearing loss. These include communication difficulties, emotional stress and effects on relationships, social life and daily routines [12,13,14,15].

The World Health Organization’s International Classification of Functioning, Disability and Health (ICF) [16] offer a valuable framework and bio-psychosocial model of health for identifying these effects [17]. It explains that an individual’s functioning depends on various factors, including their health condition, body functions and structures (such as emotional, cognitive and physical aspects), daily activities, participation in life, and contextual factors [16]. Hearing loss affects communication, which involves both AHL and SOs [17]. The ICF refers to this shared impact as “Third-Party disability”, which refers to ‘‘the study of disability and functioning of family members due to the health condition of significant others’’ [18]. This framework served as the basis of the study.

Aural rehabilitation (AR) is a broad term that includes a wide range of intervention options. A commonly accepted definition of AR is “the reduction of hearing-loss-induced deficits of function, activity, participation, and quality of life through sensory management, instruction, perceptual training, and counseling” [19]. Basura et al. [20] has published clinical practice guidelines, aiming to guide the implementation of person-centered AR for AHL. The four components of AR outlined in the guidelines are (a) Sensory management, (b) Informational counseling, (c) Perceptual training, and (d) Personal adjustment counseling. Various structured AR programs have been developed and assessed in Western contexts, predominantly in English. Some of these include Listening and Communication Enhancement (LACE) [21], Hearing Equality through Accessible Research and Solutions (HEARS) [22], Active Communication Education (ACE) [23], and Hearing and Communication Assistant for Resident Engagement (HearCARE) [24].

The above-mentioned AR programs may not completely align with the linguistic and family dynamics of the Kannada-speaking population in India. Kannada, being an official language in Southern India and the eighth most spoken language in India, with 4.37 million native speakers, accounts for approximately 3.61% of India’s total population [25]. This highlights the critical need to gather insights from stakeholders–professionals, hearing aid users, and non-hearing aid users, along with their SOs, to cross-verify the components for AR programs tailored to this population. The focus group discussion (FGD) was instrumental in this process, as it included their expertise, preferences, and needs concerning the AR. The purpose of FGD was to review the relevance and acceptability of components of AR identified in the literature to the proposed population, rather than exploring the new components, if any. Hence, we aimed to conduct one FGD for each stakeholder group (audiologists, AHL and their SOs) to obtain input regarding the context and relevance of the components of AR. With this approach, the identified components of AR for the Kannada speaking population should be practical, acceptable and effective in improving their hearing-related QoL and third-party disability of their SOs.

## 2. Materials and Methods

### 2.1. Ethical Approval

The study was conducted in accordance with the Declaration of Helsinki and was approved by an Institutional Ethics committee (IEC KMC MLR 01/2024/19, dated 18 January 2024).

### 2.2. Participants

Three FGDs were conducted, one comprising audiologists, one with AHL, and one with their SOs. Participants were recruited through purposive sampling to ensure diverse representations. The first focus group consisted of five audiologists selected from various regions in Karnataka [26,27]. Criteria for recruitment included professionals with at least a master’s degree and an experience of two years in dispensing hearing aids to AHL. The second focus group included five adults with acquired hearing loss who attended the Department of Audiology and Speech Language Pathology for audiological evaluations [26,27]. The participants were required to have a duration of hearing loss of at least six months. The AHL included both hearing aid users and non-hearing aid users. The third focus group comprised five SOs of AHL. SOs needed to have self-reported normal hearing and at least six months of experience supporting their family members with hearing loss.

### 2.3. Procedure

The moderator’s guide for the FGDs was developed on the basis of the ICF framework and clinical practice guidelines for AR [20] (provided in Appendix A). All three moderators’ guides were created to elicit experiences related to body structure and functions (hearing and communication), day-to-day activities, and participation in family, work, and social roles, as well as relevant environmental and personal factors. In addition, specific questions were included to obtain the ICF concept of third-party disability by exploring how significant others are affected by hearing loss and are involved in its rehabilitation.

The FGD for audiologists was held online using the Microsoft Teams platform [28] because of the difficulty in gathering all the professionals physically in one place. The session was moderated by the second author of the study, an Associate Professor (U.S.) with more than fourteen years of experience in the field of Audiology. The discussion was conducted in English, lasted for almost 90 min [29] and was recorded with prior consent from the participants. The FGD for AHL and their spouses was conducted separately, face-to-face within the department. The session was moderated by the first author of the study, a Research Scholar with approximately three years of expertise in the field of Audiology. The entire session was audio-recorded with prior consent from the participants. The duration of both FGDs ranged from approximately 50 to 60 min and were conducted in the Kannada language.

### 2.4. Data Analysis

The audio recorded during the focus group session was transcribed and then translated whenever required, and subsequent data analysis utilized the inductive thematic analysis method [30]. This process included several key steps: (1) familiarization with the data, (2) generation of initial codes, (3) theme identification, (4) review and refinement of themes, (5) definition and naming of themes, and (6) reporting of findings. Coding into meaning units was conducted by one researcher (G.N.S.), while a second researcher (U.S.) cross-verified 100% of the codes. Initially, 146 codes were generated from the transcripts. Twenty-one discrepancies in coding were identified. Most disagreements were resolved through discussions and review of the transcripts. In six instances, where consensus could not be reached, the third team member (K.K.) reviewed the transcripts and finalized the coding. Following refinement, overlapping codes were merged and redundant codes were removed, resulting in 118 final codes. The initial grouping of meaning units and theme identification was performed collaboratively by two researchers (G.N.S. & U.S.). The analysis was carried out using Atlas.ti software (version 25), and the findings are described following the Consolidated Criteria for Reporting Qualitative Research (COREQ). Transcripts were not given back to the participants, and they did not provide any feedback on the findings.

## 3. Results

### 3.1. Participants

The audiologists ranged in age from 36 to 39 years (Mage = 36.8 ± 1.3). The participants included three female and two male professionals who had more than 10 years of experience and were working in various settings such as government institutions, private universities with hospitals, and private clinics. Detailed information about the five AHLs is provided in Table 1. Significant others were aged 33 to 52 years (Mage = 41.6 ± 9.5).

### 3.2. Themes

Themes generated from all three FGDs are explored in detail, along with supporting quotations. Each participant was assigned a unique anonymized identifier, which is provided at the end of each quotation. Abbreviations for Audiologists (A), adults with hearing loss (AHL), and significant others (SO) are used prior to the numbers.

#### 3.2.1. Audiologists

**Figure 1:**
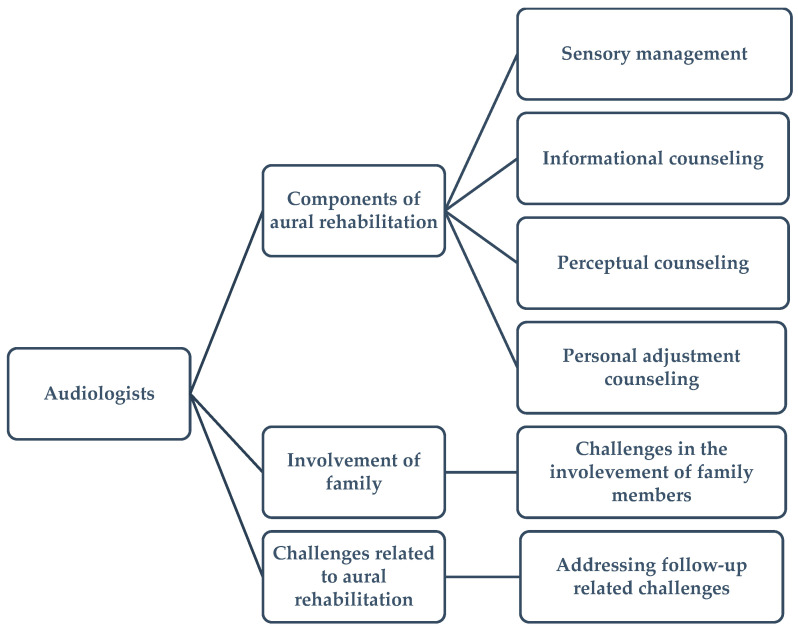
Themes and subthemes generated from the FGD involving audiologists.

Three major themes and five subthemes under the major themes were identified and are provided in Figure 1.

##### Theme 1: Components of Aural Rehabilitation

The discussions among audiologists confirmed the importance of several components including sensory management, informational counseling, perceptual training, and personal adjustment counseling. Despite this consensus, clinical practices predominantly emphasize the provision of hearing aids in conjunction with informational counseling. Conversely, perceptual training and personal adjustment counseling are less frequently implemented in clinical settings.

Subtheme 1: Sensory management

Audiologists have underscored the significance of hearing aids as the primary intervention for AHL. Following the fitting, real ear measurements are important for verifying the accuracy. Additionally, subjective assessments using validated questionnaires such as the Client Oriented Scale of Improvement (COSI) [31] and the Abbreviated Profile of Hearing Aid Benefit (APHAB) [32] help in understanding listening needs before fitting. Objective improvement metrics can be measured using the Speech Intelligibility Index (SII) or the Articulation Index (AI). For long-term users, it is crucial to evaluate and upgrade technology, including features like Bluetooth streaming. Audiologists should assess patients’ technological proficiency to ensure the effective use of these features. In challenging environments, assistive listening devices such as remote microphones or television streamers may be recommended for additional support.

*Sensory management is the first thing for any degree of hearing loss.*

(A1)

Subtheme 2: Informational counseling

Informational counseling is structured into pre-fitting and post-fitting phases, with audiologists using visual aids or pamphlets for support. Pre-fitting counseling addresses the nature and causes of hearing impairment, its impact on daily life, social interactions, work and long-term effects on cognition. It covers management options, focusing on hearing aids, including types, selection criteria, realistic expectations and the benefits of binaural hearing aids. Furthermore, AHLs and their SOs also learn communication strategies to enhance interactions. Post-fitting counseling focuses on adapting to hearing aids, offering guidance on acclimatization, care, maintenance, and troubleshooting of hearing aids for both AHL and SOs. SOs receive training to support family members and are advised to avoid fostering over-reliance, promoting independence. Finally, the significance of follow-up appointments for ongoing support and adjustment is emphasized, making this counseling important for effective AR.

*We can give a lot of informational counseling to the family members on the communication strategies they can use initially so that the client adjusts with the hearing aids at least in the initial period and there are fewer rejection rates.*

(A4)

Subtheme 3: Perceptual training

During the discussion, only a few audiologists underscored the necessity of perceptual training for AHL. For instance, patients who struggle to understand conversations in noisy environments could benefit from speech-in-noise training. Additionally, individuals with coexisting conditions such as tinnitus might find perceptual training or sound therapy beneficial for adapting to auditory challenges. The significant role of cognitive function in AHL was also noted, leading to a recommendation for cognitive training in rehabilitation. This subtheme of perceptual training was emphasized by only two out of the five audiologists, indicating a potential area for further exploration and development in AR programs.

*In the perceptual training like maybe training may not be required for everybody maybe for people with tinnitus and hearing loss who are not able to cope up just with the fitting of the hearing aid are given training so we have to make them understand how the perceptual training can also help them hearing better in the presence of noise.*

(A1)

Subtheme 4: Personal adjustment counseling

This subtheme, identified during the discussions, received limited attention from the participating audiologists. Those who addressed it underscore the critical need to consider the emotional and psychological aspects of AHL and their SOs. They highlighted that counseling should encompass empowerment and self-advocacy training, enabling patients to articulate their needs confidently and assertively. Additionally, counseling efforts should extend to SOs, offering guidance on how to adapt to and support their family members’ hearing loss. This focus on personal adjustment counseling is a fundamental component of AR, although it was discussed by only a few professionals in the study.

##### Theme 2: Involvement of Family

Audiologists have reached a consensus on the critical role of family involvement in AR, beginning at diagnosis. They highlighted the challenges families face in communicating with AHL and recommended guidance to adapt to these changes and manage interactions to avoid negative behaviors, such as shouting, anger, or frustration. Counseling should provide skills for the care, maintenance, and troubleshooting of hearing aids, particularly for older adults who may require additional support. Establishing boundaries is crucial for promoting patient independence while ensuring assistance. Educating family members about effective communication strategies is essential for enhancing interactions and overall success of AR.

*We should also look at the status of the family members or the spouse, or at least the immediate family members. Therefore, unless we address their issue, I think the whole aural rehabilitation process is not going to be, you know, we cannot trully say it is successful.*

(A2)

*Not just on the communication needs of the patient themselves, but on how much compensation is being done by the family members to ensure that they are able to communicate smoothly.*

(A4)

*It is not only about the patient for me, but also about the family members as well.*

(A5)

Subtheme: Challenges in the involvement of family members

The FGD highlighted several critical challenges concerning the involvement of family members in AR. One significant hurdle is the limited time that family members can dedicate to the rehabilitation process, which can impact the effectiveness of the support provided. Additionally, the motivation level of family members plays a crucial role in determining the success of AR. Caregiver burnout emerged as a noteworthy concern, often hindering consistent support. Age-related mobility issues among family members were also discussed, as these issues can limit their ability to assist effectively. Furthermore, in a developing country such as India, where many individuals reside in rural areas, transportation to rehabilitation centers presents a significant obstacle. These findings underscore the complexities surrounding family involvement in AR.

*So, the main challenge is going to be time. And the second thing is motivation.*

(A2)

*So, because caretaker burnout is real, we constantly cannot expect spouses or caretakers to constantly make allowances for hearing aid users.*

(A4)

##### Theme 3: Challenges Related to Aural Rehabilitation

The FGD identified several challenges pertaining to the AR of AHL, particularly within the Indian context. A significant barrier is the societal stigma associated with hearing loss and the use of hearing aids, which often leads to denial of the condition. Additionally, age-related mobility issues can hinder patients’ ability to attend rehabilitation centers physically. Transportation logistics further complicate access to these facilities. Co-morbidities related to aging and other health factors may also impact participation in rehabilitation programs. Socio-economic status plays a crucial role in the rehabilitation process, as financial constraints can prevent patients from seeking necessary care. Beyond these practical challenges, the motivation and availability of the AHL are paramount for a successful AR initiative. Furthermore, adherence to rehabilitation programs often decreases because of insufficient education and awareness among the Indian population. Audiologists also highlighted a notable lack of suitable educational resources or informational materials, such as pamphlets, that could enhance awareness about hearing loss and available interventions.

*One big challenge everyone faces is when a person is very aged, not mobile, not able to walk by themselves to come to a clinic and get a follow-up done.*

(A1)

Subtheme: Addressing follow-up related challenges

This discussion explores potential strategies to mitigate the above-mentioned challenges. For patients who face difficulties attending rehabilitation centers, scheduling home visits can be an effective solution. Additionally, enhancing the appointment scheduling process and providing timely reminders may increase attendance rates. For individuals with transportation or mobility challenges, telephone follow-ups can serve as an alternative means of support. With the advent of advanced technology, remote consultations provide opportunities for virtual troubleshooting, reprogramming of hearing aids, and addressing various rehabilitation concerns. These approaches can significantly improve access to care and facilitate ongoing support for patients with hearing loss.

*We can address these challenges through home visits. We do have home visits to assist hearing and we also fit hearing aids by doing home visits. So, when we do home visits, half of the problems of commuting and coming to a clinic or institute are cut down.*

(A1)

#### 3.2.2. Adults with Hearing Loss

**Figure 2:**
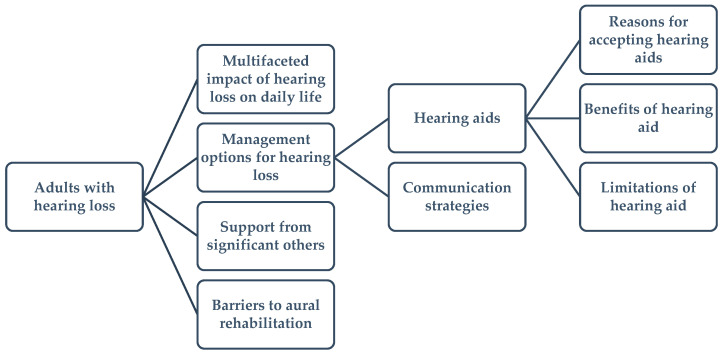
Themes and subthemes generated from the FGD involving adults with hearing loss.

Four key themes emerged from discussions with adults experiencing hearing loss, which are provided in Figure 2.

##### Theme 1: Multifaceted Impact of Hearing Loss on Daily Life

The participants discussed several challenges, including difficulties in hearing conversations from a distance, understanding speech in noisy environments, difficulty detecting warning sounds while crossing the roads, sound sensitivity (recruitment), and challenges in the workplace. Many AHLs tend to avoid social outings and hesitate to engage in conversations or request repetitions due to frustration with others’ reactions, which often leads to misunderstandings with family members and peers. The emotional consequences were significant, with feelings of insecurity, an inferiority complex, shame, social anxiety, isolation and fear of further deterioration. Reluctance to disclose hearing loss was prevalent and was influenced by societal stigma and a lack of awareness. While some were open to seeking assistance from family or friends, a few felt uncomfortable doing so. Additionally, the financial burden of hearing aids, along with repair costs, was a common concern, underscoring the profound impacts of hearing loss and the need for increased awareness and support.

*I will have to ask for repetitions when I don’t understand. Then people who are with us also get irritated. Then we feel hesitant to speak or ask for repetition.*

(AHL 3)

##### Theme 2: Management Options for Hearing Loss

Most participants had limited awareness of the available management options for hearing loss, primarily recognizing hearing aids as the sole solution. Some participants mentioned having been advised by healthcare providers to try pranayama, an ancient breathing technique, but they found it ineffective after a period of use. A few participants also reported being recommended dietary supplements that did not yield any benefits. Only a small number expressed the need for psychological counseling to help address their insecurities related to hearing loss. Overall, there was a clear lack of awareness regarding management options beyond hearing aids.

*If I use hearing aids, everything will be alright.*

(AHL 5)

*Subtheme 1: Hearing aids*

Hearing aids emerged as the most recognized management option for hearing loss among participants.

Subtheme 1.1: Reasons for accepting hearing aids

Some noted that they were forced to use them by family members, such as their spouses or children, who also faced communication difficulties stemming from the participants’ hearing loss. Others indicated a proactive choice to use hearing aids in order to improve their hearing capabilities and enhance communication.

Subtheme 1.2: Benefits of hearing aids

Participants who used hearing aids reported several perceived advantages—an improvement in hearing ability, which facilitated better understanding while watching television and improved communication during phone conversations. Overall, the level of satisfaction among hearing aid users was high.

Subtheme 1.3: Limitations of hearing aid

In addition to the noted benefits, participants highlighted certain limitations of hearing aids. Some users experienced a reduction in speech clarity, particularly in noisy environments. Many reported that hearing aids often amplify sounds to an uncomfortable level, particularly in challenging listening situations. Additionally, participants mentioned that they experienced distortions, which contributed to difficulties in understanding speech.

*Subtheme 2: Communication strategies*

The participants displayed some awareness of useful communication strategies. Many reported the importance of facing the speaker during conversations to assist with speechreading. Some expressed hesitation in asking for repetitions, leading them to resort to guesswork when they did not understand parts of the conversation. These findings suggest that many AHL may not be fully informed about effective communication strategies, highlighting the need for communication strategy training in AR practices.

##### Theme 3: Support from Significant Others

The AHLs identified several forms of support they expect from SOs. A primary expectation is that family members will exhibit high patience when addressing communication challenges. Effective communication strategies, including speaking from a close distance and the willingness to repeat information as needed were also emphasized. This feedback shows that it is important for SOs to communicate clearly and adapt to the needs of individuals with hearing loss.

*Family members should have good patience. They also get irritated when we ask them for repetition.*

(AHL 3)

##### Theme 4: Barriers to Aural Rehabilitation

Age-related factors, including vision impairments, were noted to hinder attendance at rehabilitation centers. Additionally, societal stigma associated with hearing loss and hearing aids, along with negative comments from peers, contributed to feelings of shame that further obstructed the rehabilitation process. Participants reported difficulties in decision-making when confronted with multiple treatment options, with some expressing concerns about the frequent need for repairs of their hearing aids, leading to financial strain. Furthermore, several individuals used their hearing aids inconsistently, often relying on only one device despite having two. Participants were unaware of alternative management options for hearing loss beyond hearing aids and exhibited reluctance to participate in AR programs. This highlights a substantial gap in awareness regarding hearing loss and its treatment, emphasizing the critical need for educational initiatives to inform individuals about available rehabilitation options.

*I feel ashamed to go out and face people.*

(AHL 1)

#### 3.2.3. Significant Others of Adults with Hearing Loss

**Figure 3:**
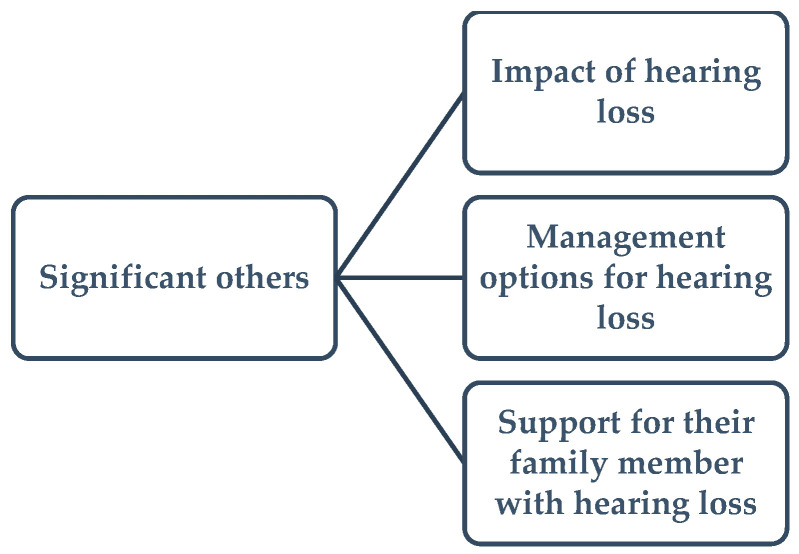
Themes and subthemes generated from the FGD involving significant others of adults with hearing loss.

In the FGD involving SOs of AHL, three primary themes emerged as provided in Figure 3.

##### Theme 1: Impact of Hearing Loss

Difficulty in communication due to the presence of hearing loss was the major difficulty faced by the SOs. They have to speak louder than the usual volume for the AHL to understand. Then, the SOs also had to frequently check on family members with hearing loss to see if they truly understood or they were simply pretending to understand.

*Difficulties one, we have to speak a little louder. Then another, sometimes they, even if they have not heard, they will just respond. So, I have to check again. I’ll ask her, what did I tell her?*

(SO 2)

##### Theme 2: Management Options for Hearing Loss

In line with the perceptions of AHL, participants who were SOs also expressed the belief that the provision of hearing aids alone was adequate for management. The SOs recommended the integration of counseling sessions specifically designed to support them in coping with the challenges associated with their family members’ hearing loss. They emphasized the need for informative counseling to enhance their understanding of the underlying causes and progression of hearing loss. Overall, there was a notable deficiency in awareness regarding comprehensive management options among SOs, indicating a need for educational initiatives aimed at broadening their knowledge and facilitating better support strategies for AHL.

*I think general awareness is not there. Because if I noticed, like when my relatives were old, many people had hearing loss. But they don’t use a hearing aid. They just ask the other person to repeat again and again. And I think generally, they feel that hearing aid is some stigma.*

(SO 2)

*Subtheme 1: Hearing aids*

During the discussions concerning the role of SOs in facilitating the acceptance of hearing aids by family members with hearing loss, several key motivations were identified. SOs primarily aimed to minimize misunderstandings and enhance communication with their relatives with hearing loss. Additionally, they expressed concerns related to the well-being of the individual with hearing loss, highlighting their desire for that person to maintain social engagement and ensure personal safety. These findings underscore the important role of family dynamics in the acceptance and use of hearing aids.

*Subtheme 2: Communication strategies*

The SOs discussed various communication strategies they employ to enhance communication—speaking loudly, repeating information, positioning themselves to face the person with hearing loss while speaking, directing speech toward the better ear, speaking from a nearby distance, and minimizing background noise by, for instance, turning off fans before conversations. Additionally, some participants noted other strategies such as gaining the attention of the individual with hearing loss before initiating conversation, cross-checking if the person understood the communicated message, and, when necessary, writing down key information to aid understanding in challenging situations. These insights highlight the adaptive strategies that SOs implement to facilitate effective communication with the AHL.

##### Theme 3: Support for Their Family Members with Hearing Loss

Significant others emphasize empathy and understanding, creating a supportive environment for family members with hearing loss. They recognize the importance of educating these individuals about hearing loss as a natural part of aging. Additionally, SOs use various communication strategies to bridge the communication gap and ensure their family members feel included in conversations. Overall, the role of SOs is vital in promoting an accommodating environment for AHL.

*So, I think one thing is we need to explain to him that this is a natural aging process and make him accept the reality.*

(SO 3)

In addition to the thematic analysis, a co-occurrence analysis was conducted on data collected from AHL and their SOs. This analysis revealed an interesting finding. Notably, those adults who expressed satisfaction with their hearing aids stated that they considered them sufficient for managing their hearing loss without the need for additional interventions. Given the limited sample size, these aspects warrant further exploration in a larger sample.

## 4. Discussion

This study explores the perspectives and experiences of audiologists, AHLs, and their SOs regarding the components of AR for Kannada-speaking people in the southern part of India. Importantly, this study also highlights the perspectives and needs of SOs related to aural rehabilitation, which have not been extensively examined in either Western or Indian contexts. The findings of this study provide valuable insights into how AR is perceived and practiced in this population, and how these perspectives align with or differ from those in the Western literature/context.

Overall, the findings highlight multiple components of AR proposed in the literature by Boothroyd [19] and Basura et al. [20], which include sensory management, informational counseling, perceptual training and personal adjustment counseling. Audiologists advocated for a comprehensive approach to AR, that extends beyond the provision of hearing aids. In contrast, a significant number of AHLs and their SOs expressed the belief that hearing aids alone were adequate. These findings contrast with Archana et al. [33], who reported that merely fitting hearing aids was insufficient among adults and elderly hearing aid users and highlighted the importance of supplemental auditory training to enhance communication ability. This difference may stem from a limited awareness of the broader scope of AR among the participants and the potential benefits of the additional components in enhancing their rehabilitation experience. The study revealed various communication difficulties experienced by both AHLs and their SOs. This aligns with the literature on the diverse impacts of hearing loss on adults and their SOs [34,35]. Together, these findings highlight the multifaceted nature of hearing loss and strengthen the need for AR programs that focus on not just auditory abilities, but also emotional, social and practical challenges.

### 4.1. Aural Rehabilitation of Adults with Hearing Loss

The four components of AR identified in the Basura et al. [20] study was discussed in FGDs led by audiologists in the current study. Sensory management emerged as the primary focus, particularly the utilization of hearing aids, which was emphasized by audiologists, AHL, and their SOs. While various options exist in sensory management, hearing aids remain the predominant intervention, as supported by Kiessling et al. [36] and reaffirmed in this study. Audiologists highlighted the importance of informational counseling alongside hearing aid fitting to enhance outcomes, while SOs expressed a need for such counseling to better understand the etiology and progression of hearing loss. Such counseling sessions are essential for helping family members manage the challenges associated with their family member’s hearing loss effectively. While perceptual training was not widely recognized as essential, AHL expressed a desire for psychological counseling to address insecurities related to their condition, aligning with the concept of personal adjustment counseling. A few audiologists have also emphasized personal adjustment counseling. This need for holistic support is corroborated by Makhoba and Joseph [37], who reported that a significant proportion of audiologists (81.4%) provided hearing aids, followed by communication strategies training (69.8%) and informational counseling (79.8%). These findings suggest that clinical practice predominantly focuses on amplification and informational counseling, with less attention given to perceptual training and personal adjustment counseling. Sweetow and Palmer [38] highlighted that many audiologists primarily concentrate on restoring audibility through amplification, indicating a need for a comprehensive AR approach, encompassing sensory management, psychological and educational support. Audiologists emphasize the need to develop customized comprehensive AR plans tailored to the specific needs of AHL. Several studies advocate adapting AR strategies to meet each patient’s needs [39,40].

However, the present study identified several challenges related to the AR of AHLs and their SOs, notably the societal stigma associated with hearing loss and the use of hearing aids in India. This stigma often leads to denial among AHLs, as negative peer comments and societal attitudes foster feelings of shame that hinder rehabilitation. The literature indicates that hearing loss and hearing aids are perceived as signs of aging or vulnerability, leading many people to avoid them due to fear of judgment or negative labeling. The stigma manifests as feelings of shame, anxiety over social rejection, and concerns about personal appearance [41,42,43]. Additionally, negative stereotypes regarding hearing loss and hearing aids significantly impact the willingness to disclose hearing loss and utilize hearing aids [44]. These findings highlight the need for AR programs to incorporate counseling to help individuals cope with stigma and involve relatives and partners of AHLin discussions. Interestingly, Bennett et al. [45] examined clinicians’ perspectives on group audiological rehabilitation, identifying barriers such as infrequent recommendations, insufficient prioritization in workflows, and unclear delivery plans. Organizational challenges included the unavailability of services, a lack of support, and inadequate resources and funding. Clinicians expressed a need for resources such as lesson plans and training videos, highlighting the importance of a standardized AR module with clear instructions and additional training for clinicians. Addressing these multifaceted barriers is essential for improving patient outcomes. Educating patients and their families about management options beyond hearing aids and developing supportive resources will empower clinicians and create a more inclusive rehabilitation environment for AHL.

### 4.2. Involvement of Family in Aural Rehabilitation

The involvement of family members is vital in AR. Research highlights the advantages of family participation during appointments, including enhanced shared understanding, increased support, and more comprehensive intervention plans [46,47,48]. Meyer et al. [48] emphasized that audiologists should cultivate collaborative partnerships with family members to increase participation in the rehabilitation process, ultimately leading to improved outcomes for AHL [49,50]. These findings highlight the necessity of developing a structured AR program for SOs, coupled with adequate training to facilitate their involvement effectively.

However, Meyer et al. [48] reported that low attendance rates among family members persist because of barriers such as time constraints and misconceptions about hearing aids. Similar challenges, including a lack of awareness about hearing loss and available interventions, motivation, caregiver burnout, age-related mobility issues, and transportation issues were discussed in this study. Addressing these barriers is crucial for improving family involvement in AR. By fostering awareness and providing solutions for challenges such as transportation and caregiver burnout, participation rates can improve, positively impacting outcomes for AHL. A collaborative approach that includes family members can significantly enrich the rehabilitation experience and promote better long-term acceptance of hearing aids.

### 4.3. Proposed AR for the Kannada-Speaking Population

While the main elements of AR found in Western frameworks were mostly relevant to the Kannada-speaking community in this study, several contextual factors suggest the need for adaptation to the local sociocultural environment. Specifically, there is a limited understanding of AR options beyond hearing aids, and a strong dependence on family support, financial challenges, and stigma related to hearing loss and hearing aids. To address these challenges, it is essential to conduct more awareness programs that educate AHLs and their SOs. These programs should focus on several AR options, highlighting that hearing aids are just one part of a broader spectrum of AR services. An outline of the proposed AR program customized for the local population is provided below:

- Sensory management—includes the selection and fitting of hearing aids/assistive listening devices, and the care, maintenance and troubleshooting of hearing aids.
- Informational counseling (both AHL and SOs)—includes enhancing the understanding of hearing loss, management options and realistic expectations from hearing aids.
- Communication strategies training (both AHL and SOs)—includes teaching various communication strategies to facilitate effective communication.
- Clear speech training (for SOs)—includes strategies for SOs to use when communicating with an AHL.
- Personal adjustment counseling—includes addressing emotional responses to hearing loss, reducing the stigma and promoting coping strategies.

## 5. Limitations

Several limitations of the present study should be noted. First, the sample size was relatively small, consisting of only 15 participants, which may limit the generalizability of the findings. A larger sample could enhance the robustness and applicability of the results to a broader population. Second, only one round of FGD was conducted, which may have prevented the achievement of data saturation. Third, there is a paucity of information regarding participant characteristics, such as education level, lifestyle, and quality of life which may have influenced the diversity of perspectives captured in the study.

## 6. Conclusions

In conclusion, this study highlights the importance of a comprehensive multi-component AR program for AHLs and their SOs. By incorporating multi-component AR, the program aims to enhance the overall rehabilitation experience. The inclusion of SOs will enhance their understanding of hearing loss and foster a supportive atmosphere, ultimately promoting the acceptance of hearing loss. This holistic approach not only addresses the functional challenges of hearing loss but also empowers individuals to communicate more effectively in their daily lives.

## Figures and Tables

**Table 1:** Detailed information about the five AHLs.

	AHL 1		AHL 2		AHL 3		AHL 4		AHL 5	
Age (years)	62		65		63		77		84	
Gender	Male		Female		Female		Male		Female	
Laterality	Bilateral		Bilateral		Bilateral		Bilateral		Bilateral	
Duration of HL	5–6 years		4–5 years		3 years		4 years		1.5–2 years	
PTA (dBHL)	R: 70	L: 67.5	R: 83.75	L: 87.5	R: >90	L: 40	R: 76.25	L: 63.75	R: 50	L: 85
Type of hearing loss in both ears	Mixed		Sensorineural		Mixed		Sensorineural		Sensorineural	
SIS (%)	R: 40	L: 60	R: 30	L: 20	R: CNT	L: 85	R: 45	L: 65	R: 70	L: 30
Hearing aid usage	Yes, Bilateral		Yes, Unilateral		Yes, Bilateral		No		No	
Occupation	Shopkeeper		Home maker		Retired bank employee		Clerk		Home maker	
Relationship of SO with AHL	Wife		Daughter-in-law		Son		Son		Son	

Note: AHL—Adult with hearing loss, PTA—Pure tone average, SIS—Speech identification scores, SO—Significant other, CNT—Could not test, R—Right ear, and L—Left ear.

## Data Availability

The data related to the results of this study can be obtained from the corresponding author upon a reasonable request.

## Abbreviations

The following abbreviations are used in this manuscript:

AR	Aural rehabilitation
AHL	Adults with hearing loss
SOs	Significant others
FGD	Focus group discussion

## Appendix A

**Moderator’s guide for audiologists, adults with hearing loss and their significant others is provided. Each section of the guide has been mapped to the ICF components and third-party disability.**

**Audiologist**

*Domain of interest: Current Practices and Perspectives (Body functions, activities/participation issues)*

1. What are the current practices and approaches you use in auditory rehabilitation for adults with hearing loss and their spouses?
2. What else is done apart from providing hearing aids? (Probe: adults who use HA, adults who do not use HA, spouses of adults who use HA, spouses of adults who do not use HA)

*Domain of interest: Identification of key components (Body functions, activities/participation issues, emotional functions, Third-party disability)*

3. In your opinion, what are the essential components that should be included in an auditory rehabilitation program for adults with hearing loss (Probe: sensory management, informational counselling, personal adjustment counselling, perceptual training)
4. How do you prioritize these components based on the severity of hearing loss and individual needs?
5. How do you prioritize these components for
  - Non-hearing aid users
  - Less frequent hearing aid users
  - Regular hearing aid users
6. How important is it to address the emotional and psychological aspects of hearing loss in rehabilitation? (Probe: adults with hearing loss, spouses of adults with hearing loss)
7. In your opinion, what are the essential components that should be included in an auditory rehabilitation program for spouses of adults with hearing loss?

*Domain of interest: Involvement of spouse and family members (Third-party disability and environmental factors)*

8. What role do spouses and family members play in the auditory rehabilitation process?
9. How can spouses and family members be involved in the auditory rehabilitation process?
10. What strategies can be used to educate and support spouses in understanding and coping with their partner’s hearing loss?
11. What are the possible challenges that can be encountered while engaging spouses and family members, and the possible solution to overcome them?

*Domain of interest: Challenges (Contextual factors—environmental and personal factors)*

12. What are the possible challenges that can be faced while delivering effective auditory rehabilitation for adults with hearing loss and their spouses?
13. How can we address barriers to participation and adherence to rehabilitation programs?
14. Is there anything else you would like to add or discuss regarding auditory rehabilitation for adults with hearing loss and their spouses?

**Adults with hearing loss**

*Domain of interest: Personal Experiences (Body functions, activities/participation issues)*

1. What do you think are the difficulties faced by individuals with hearing loss?
2. How do you think hearing loss will impact the daily life of individuals with hearing loss? (Spouse/family members, work and social life)
3. What do you think are the advantages of using hearing aids? (Only for HA user)
4. What do you think are the limitations of hearing aids? (Only for HA user)
  - Please elaborate on how can one cope-up with those limitations?
5. What specific challenges can be faced in managing hearing loss, and how can it be addressed? (For non-HA user)
6. Do you think any form of training will be useful for hearing better?
  - If yes, what kind of training can be expected? / Training is required to help with what aspects related to listening.
  - If no, why do you think so?
7. Apart from using hearing aids do you think any other form of training would be helpful for hearing better? (Only for HA user)
  - If yes, what kind of training can be expected? / Training is required to help with what aspects related to listening.
  - If no, why do you think so?
8. What additional services do you think are required to cope up with the difficulties related to hearing loss? (For both HA and non-HA user)

*Domain of interest: Needs and Preferences (Environmental factors)*

9. What are the services that can be expected after the diagnosis of hearing loss?

*Domain of interest: Spousal and Family Support (Environmental factors)*

10. How can spouse or family members supported the journey with hearing loss?
11. In what ways do you think spouses and family members can be more involved in the treatment process?

*Domain of interest: Challenges and Barriers to Participation (Contextual factors—environmental and personal factors)*

12. What factors can influence decision to accept hearing aids?
13. What are the reasons for non-acceptance of hearing aid?
13. What are the challenges that can be faced related to acceptance of hearing aid?
14. According to you what are the societal stigma that are faced during initial period of hearing aid use?

**Significant others**

*Domain of interest: Spousal Experiences and Perspectives (Third-party disability, Body functions, activities/participation issues, contextual factors—environmental and personal factors)*

1. What do you think are the difficulties experienced by spouses of someone with hearing loss?
2. How will one’s spouse’s hearing loss impact their relationship and daily interactions?
3. What are the most significant challenges faced by spouse of someone with hearing loss?
4. What can be the role of a spouse in making the spouse accept hearing aids? (For spouses of HA user)
5. What can be the role of a spouse in spouse not accepting hearing aids? (For spouses of non HA users)
6. What strategies can be used to communicate effectively with the spouse?
7. What additional services do you think are required to help one cope up with the difficulties related to their spouse’s hearing loss? (For both HA and non-HA user)
8. Do you think any kind of domestic violence happens due to the presence of hearing loss in spouses? If yes, elaborate.

*Domain of interest: Needs and Expectations (Environmental factors)*

9. What do you perceive as the most important treatment related needs of individuals with hearing loss and their spouses?
10. What components or services do you believe should be included in the treatment to support both individuals with hearing loss?
11. What components or services do you believe should be included in the treatment to support spouses of individuals with hearing loss?

*Domain of interest: Communication Strategies and Support (Activities and participation issues, third-party disability)*

12. In what ways do you think treatment for hearing loss can enhance communication skills and support both individuals with hearing loss and their spouses?

*Domain of interest: Understanding and Coping with Hearing Loss (Personal factors, emotional functions)*

13. How has your understanding of hearing loss evolved since your spouse was diagnosed?
14. What emotional and psychological impact can one’s spouse’s hearing loss have on their relationship?
15. What support mechanisms or coping strategies can be helpful in managing the challenges associated with your spouse’s hearing loss?

*Domain of interest: Involvement in Rehabilitation Process (Environmental factors)*

16. What role do you believe spouses should play in supporting their partners during the treatment for hearing loss?

*Domain of interest: Impact on Relationships and Quality of Life (Activities/participation issues)*

17. How can one’s spouse’s usage of hearing aids affect their relationship and quality of life? (only for HA users)
